# Support Systems and Limitations in Therapeutic Abortion Care by the Gynecologist-Obstetrician of Public Hospitals in Peru

**DOI:** 10.1055/s-0042-1746198

**Published:** 2022-07-12

**Authors:** Juan Matzumura, Hugo Gutierrez-Crespo, Enrique Guevara, Luis Meza, Mauricio La Rosa

**Affiliations:** 1 Department of Obstetrics and Gynecology. Universidad Nacional Mayor de San Marcos, Lima, Peru; 2 Department of Obstetrics and Gynecology. Instituto Nacional Materno Infantil, Lima, Peru; 3 Department of Obstetrics and Gynecology. Universidad Peruana Cayetano Heredia, Lima, Peru; 4 Department of Obstetrics and Gynecology. Division of Maternal Fetal Medicine. University of Texas Medical Branch, Texas, United States

**Keywords:** abortions, therapeutic, hospitals, training, participants, abortos, terapêutico, hospitais, treinamento, participantes

## Abstract

**Objective** To identify the barriers to provide to women and adequately train physicians on therapeutic abortions in public hospitals in Peru.

**Methods** Descriptive cross-sectional survey-based study. We invited 400 obstetrics and gynecology specialists from 7 academic public hospitals in Lima and 8 from other regions of Peru. Expert judges validated the survey.

**Results** We collected survey results from 160 participants that met the inclusion criteria. Of those, 63.7% stated that the hospital where they work does not offer abortion training. Most of the participants consider that the position of the Peruvian government regarding therapeutic abortion is indifferent or deficient. The major limitations to provide therapeutic abortions included Peruvian law (53.8%), hospital policies (18.8%), and lack of experts (10.6%).

**Conclusion** Most surveyed physicians supported therapeutic abortions and showed interest in improving their skills. However, not all hospitals offer training and education. The limited knowledge of the physicians regarding the law and institutional policies, as well as fear of ethical, legal, and religious repercussions, were the main barriers for providing abortions.

## Resumo

**Objetivo** Identificar as barreiras para oferecer às mulheres e capacitar adequadamente os médicos sobre abortos terapêuticos nos hospitais públicos do Peru.

**Métodos** Estudo descritivo transversal baseado em inquérito. Convidamos 400 especialistas em obstetrícia e ginecologia de 7 hospitais públicos acadêmicos de Lima e 8 de outras regiões do Peru. Juízes especialistas validaram a pesquisa.

**Resultados** Coletamos os resultados da pesquisa de 160 participantes que atenderam aos critérios de inclusão. Destes, 63,7% afirmaram que o hospital onde trabalham não oferece treinamento sobre aborto. A maioria dos participantes considera que a posição do governo peruano em relação ao aborto terapêutico é indiferente ou deficiente. As principais limitações para fornecer abortos terapêuticos incluem a lei peruana (53,8%), políticas hospitalares (18,8%) e falta de especialistas (10,6%).

**Conclusão** A maioria dos médicos pesquisados apoiava o aborto terapêutico e demonstrava interesse em aprimorar suas habilidades. No entanto, nem todos os hospitais oferecem treinamento e educação. O conhecimento limitado dos médicos sobre a lei e as políticas institucionais, além do medo de repercussões éticas, legais e religiosas, foram as principais barreiras para a realização do aborto.

## Introduction

There are 73 million abortions reported worldwide every year. Approximately 45% of these are considered unsafe. In developing countries, this percentage increases to 56%. 1 In Latin America, 10 to 16% of maternal deaths are caused by unsafe abortions. 2

In developing countries, suboptimal access to abortion services is a serious problem. Women from low socioeconomic groups and other vulnerable women are disproportionately affected by the lack of information and lack of access to family planning services. 3 Therapeutic abortion must be aligned within a context of respect for sexual and reproductive rights, a fundamental part of human rights.

In Peru, “an abortion can be performed by a doctor with the pregnant woman's consent when it is the only way to save the patient's life or to avoid a serious long-term illness in her health.” 4 Despite the progress made by approving the national guideline for therapeutic abortion, women still experience inadequate access to this service. This inadequate access results in high rates of maternal mortality. 4 5 Annually, ∼ 376,000 unsafe abortions are performed in this country. 6 7

There have been several initiatives to promote the use of guidelines for therapeutic abortion and to provide specialized training to doctors. 8 Nevertheless, there are still significant gaps. Training in the management of therapeutic abortions is not routine in several residency programs in the United States. The inappropriate methodology used, the absence of simulators, and limited legal support for the institutions, also limit training. 9 10 Turk et al. 9 performed a survey of residency program directors around the United States to describe their perspective of support for and resistance to abortion training. Almost 75% of them reported at least some institutional or government restriction, with an average of 3 types of restrictions. They reported that hospital policy restrictions were common, followed by state law restrictions. 9

In 2016, Távara Orozco et al. 8 reported the status of safe therapeutic abortion in Peru based on interviews and data collection from 10 hospitals. They found that the rate of therapeutic abortions was still low, with lethal fetal abnormalities being the most common indication. 8

There is no data on the main barriers to adequate training of specialists in the management of therapeutic abortion care in Peru. Our objective is to identify the barriers to provide adequate physician training and to perform therapeutic abortions for women in public hospitals in Peru.

## Methods

We performed a descriptive cross-sectional survey-based study. We invited 400 participants, obstetrics and gynecology specialists from 7 academic public hospitals in Lima and 8 from other regions of Peru. We included obstetrics and gynecology specialists that worked in these 15 hospitals, and we excluded participants who were not routinely assigned to clinical duties or did not complete the survey. We developed our survey based on previously published surveys. 8 9 Our survey evaluated the support systems and limitations for the training and performance of therapeutic abortions. It consisted of a total of 43 questions.

The validation of the instrument was performed in two phases: content validity followed by instrument reliability phase. Six Peruvian experts performed the content validity. They had to meet the following criteria: work experience in the subject, original research on this subject, and have an academic master's or doctor's degree. The concordance index, according to the Kappa index, was 0.61. For the instrument reliability phase, we performed a pilot test with the participation of 30 gynecologists. The total reliability was 0.77.

The questionnaires were sent to the participants by email and reminder phone calls from May to November 2020. The data processing and analysis were performed using estimates to calculate absolute and relative frequencies using IBM SPSS Statistics for Windows, version 22 (IBM Corp., Armonk, NY, USA).

The Faculty of Medicine of the Universidad Nacional Mayor de San Marcos Research Ethics Committee approved the research project.

## Results

We enrolled 160 participants who completed the survey and met the inclusion criteria. The characteristics of the participants are shown in Table 1 . Almost half of the participants reported that their hospital did not provide therapeutic abortions, but > 80% support the idea of this procedure and thought it should be provided.

**Table 1:** Characteristics of the Participants

Variables	*n*	%
Age (years old) (mean ± SD)	46.8 (±12)	
Male	109	68.1
Female	51	31.9
Married	107	66.9
Single	41	25.6
Divorced	9	5.6
Widow	3	1.9
Hospital region		
Lima	120	75
Other region	40	25
Religion		
Catholic	141	88.1
None	11	6.9
Other	8	5
Position		
Faculty	141	88.1
Department director	15	9.4
Department chairman	4	2.5
Does your hospital provide therapeutic abortion?		
No	76	47.5
Yes	84	52.5
Do you think that therapeutic abortions should be provided?		
No	28	17.5
Yes	132	82.5

Table 2 described the barriers identified by the participants to train and provide therapeutic abortion at their institution.

**Table 2:** Barriers to train physicians and to provide therapeutic abortion

	*n*	%
**Training in therapeutic abortion is limited as a result of:**		
Peruvian law	73	45.6
Institutional policies	53	33.1
No relationship with an institution that provides abortion	23	14.4
Lack of medications/equipment	11	6.9
**Providing therapeutic abortion is limited as a result of:**		
Peruvian state law	80	50.0
Institutional policies	43	26.8
No relationship with an institution that provides another type of abortion	24	15.0
Lack of expert physicians	13	8.1

**Fig. 1:**
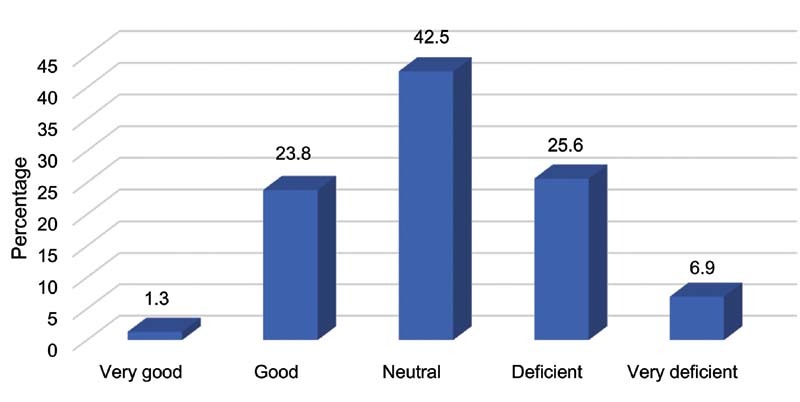
Position of the Peruvian government regarding therapeutic abortion.

Most of the participants consider that the position of the Peruvian government' regarding therapeutic abortion is indifferent or deficient ( Fig. 1 ).

Regarding training at their institution, 63.7% of the respondents stated that the hospital does not offer abortion training. Also, 46.9% reported that the training is performed in other institutions, such as scientific societies, universities, or private institutions. Table 3 describes the levels of support at their institution for training in therapeutic abortion.

**Table 3:** Level of support for training

	Lot of support		Support		Neutral		Limitations		Lot of limitations		None	
	n	%	n	%	n	%	n	%	n	%	n	%
Department leadership	12	7.5	50	31.3	47	29.4	28	17.5	16	10	7	4.4
Hospital director	9	5.6	33	20.6	70	43.8	22	13.8	16	10	10	6.3
Nurses	14	8.8	53	33.1	61	38.1	9	5.6	12	7.5	11	6.9
Anesthesiologist	5	3.1	40	25	61	38.1	25	15.6	19	11.9	10	6.3
Medical staff and equipment	18	11.3	52	32.5	42	26.3	25	15.6	13	8.1	10	6.3
Interaction with other specialties	8	5	60	37.5	53	33.1	19	11.9	10	6.3	10	6.3
Residents	49	30.6	52	32.5	37	23.1	12	7.5	9	5.6	1	0.6

More than half of the participants (56.3%) thought that abortion training should be integrated into the residency program, while 20% thought it should be part of family planning rotation. Two-thirds had availability for abortion training 1 to 3 days per week, and 22.5% between 4 and 6 days per week. The personal reasons not to participate in therapeutic abortion training were religious reasons in 17.5% and to avoid legal problems in 8.1%.

**Fig. 2:**
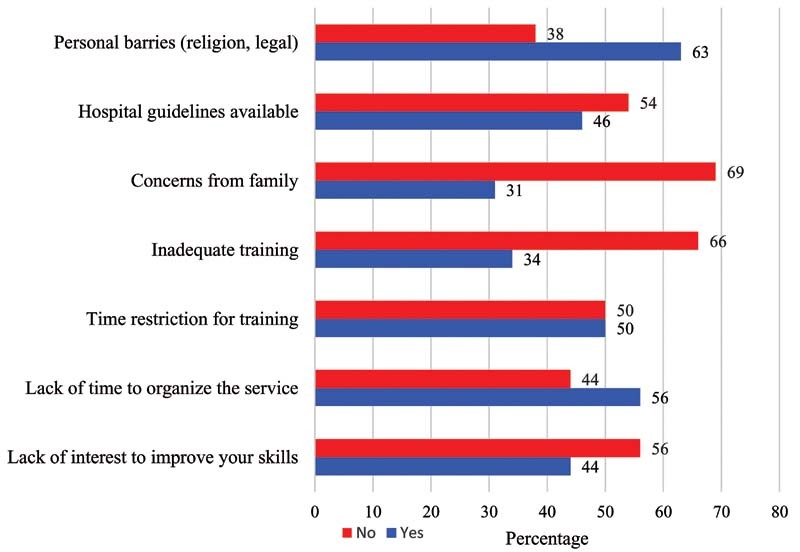
Internal barriers to provide therapeutic abortions.

**Fig. 3:**
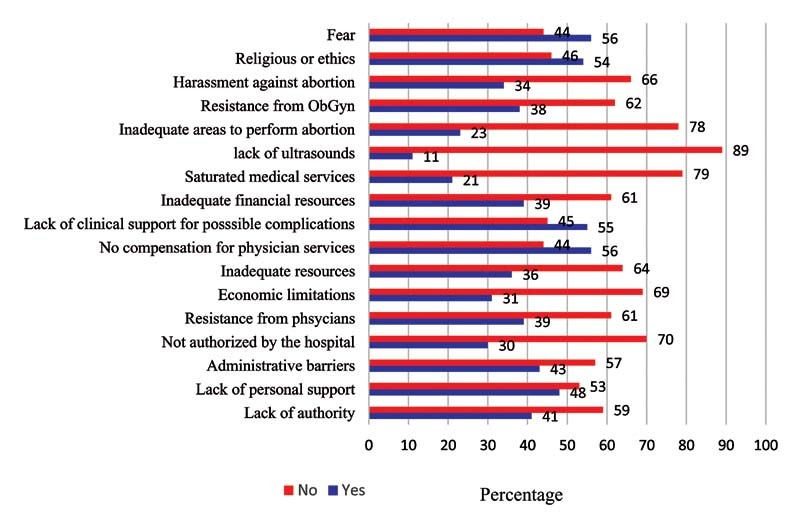
External barriers to provide therapeutic abortions.

Almost half of the participants (45%) did not receive training on abortions, 10% received training only in early failed pregnancies, and 45% received training for the management of therapeutic abortions. Almost half of the physicians (44.4%) stated that they did not perform any therapeutic abortions during residency, and only 16.9% did > 10 procedures. On the other hand, 85% stated that they had competencies for the management of abortion complications. The major barriers to providing therapeutic abortions included Peruvian law (53.8%), hospital policies (18.8%), and lack of experts (10.6%). Figures 2 and 3 describe the internal and external barriers to adequately incorporate therapeutic abortion services in their institution.

Regarding conferences that provide wellbeing resources for physicians who perform abortion, 30.6% reported that they participate once every year, while 36.3% more than once per year. The remaining did not participate in such sessions during the last years. A total of 40.6% of the participants were unaware of tools to handle emotions during and after performing therapeutic abortions.

## Discussion

To improve women's health, women's rights, and health promotion, interventions should be supported. 5

Our study showed that almost half of the specialists do not provide therapeutic abortions at their institution, although most of them support the idea of therapeutic abortion care. Access to safe abortion is crucial in the care of women's health. 11 In Latin America, each county has different laws; some limit access to safe abortion, while others make this procedure widely available for their population. 12 13 To provide safe abortion to a population, the availability of a significant number of institutions and doctors with training in this service is required. 14 The majority of physicians report limited exposure to therapeutic abortion during residency training. The lack of doctors trained in performing abortions is a problem described not only in Peru. Prior studies have reported limited access to abortions in obstetrics and gynecology training programs. 15 16 The lack of doctors trained for this procedure leads to limited or no access to safe abortion. This lack of access can lead to clandestine abortions or pregnancies carried to term despite the risk they may pose to women.

For example, in the United States, most abortions performed occur in nonacademic institutions, limiting the exposure of residents to these types of procedures. Academic institutions in that country must make different efforts to ensure the exposure of their residents to training in safe abortion. 17

As in other countries, legal regulations are one of the main barriers that limit the exposure to this procedure during specialty training. These legislative barriers are pronounced in training centers, which are public hospitals with government funding. Also, many academic centers require procedures that make the abortion process difficult; for example, consents that must be signed a few days before the procedure can be performed. These common barriers to providing safe therapeutic abortion in academic centers are consistent with the responses of the participants in our study.

In a study published by Freedman et al., 18 most doctors who wanted to provide abortion services to their community did not perform it, mainly due to legal barriers or to the institution where they worked. A study in Latin America showed that most doctors who provided services in public hospitals were not aware of the grounds on which abortion is not punishable. In this study, > 60% favored decriminalizing abortion, while only 1 in 5 had performed a therapeutic abortion in their medical practice. 19 One study in Brazil, where it is legal to perform abortions in the case of rape based on a woman's statement, showed that 82% of the physicians required police reports or judicial authorization. This requirement is a major barrier for these women to access safe abortions. 20

Access to therapeutic abortions in public institutions in Peru is limited, with just a few public hospitals providing this service. A study published in 2016 reported that in the 10 hospitals where this procedure is performed in Lima, only 257 procedures were performed in the previous 5 years. 8 A survey conducted with doctors from public hospitals in Lima showed that 44% of them did not agree with some of these legal limitations since they violate the right to doctor-patient confidentiality. 21

The impact of religion on access to training in therapeutic abortion has also been described. The fact that the institution is associated with a religious entity limits the ability to train residents in therapeutic abortion. 2 22 In our study, > 80% of the participants considered themselves catholic. However, < 20% of the participants reported that religion was a reason for not participating in therapeutic abortion training.

A recent study by Turk et al. 9 showed that the most common constraints to physician training identified by directors of residency programs in the United States included institutional or legal policies. The directors of programs that included this training as an integral part of the residency identified fewer restrictions than the directors of programs where they did not train in abortions. 9

More than 60% of the participants reported that the hospital where they practice does not offer training in therapeutic abortion, and < 50% have trained under the responsibility of another institution. On the other hand, almost 50% of the participants did not carry out a therapeutic abortion during their training, while only 17% performed > 10 procedures. The model of inclusion of abortion training during medical residency has an important impact.

The American College of Obstetricians and Gynecologists (ACOG) 14 reports three types of abortion training models in gynecology and obstetrics residencies in the United States. The first is known as “opt-out,” in which the academic center has an abortion program integrated into its curriculum. It is standard for residents to regularly perform this procedure, except for residents who opt out due to religious or moral objections. The second is the “opt-in,” in which the academic center provides training only if the resident requests to be trained in that procedure. And the third type of residency is that without abortion training. Turk et al. 15 demonstrated that residents who were trained in “opt-out” residency programs had a greater number of abortions, greater exposure to abortion procedures, and felt more comfortable in their abilities to perform this procedure. On the other hand, residents of residency programs of the “opt-out” type had the same results as residents of hospitals where this training was not performed. 15 Other studies have shown that residents graduated from “opt-out” training feel more confident in their abilities, not only to provide abortions, but also to manage other procedures and counseling in gynecology and obstetrics. 22 23 24

The training of residents in therapeutic abortion should be comprehensive and should include training in patient counseling, 1 ^st^ -trimester ultrasound, pain management, cervical dilation, as well as medical and surgical management. 14 Many studies have shown that graduates of training centers where family planning, including abortion, was an integral part of the program, have greater skill in handling not only the procedure, but also all the other aforementioned aspects. 15 21 22 25

It is crucial to be able to make changes to improve the training of physicians in family planning, including therapeutic abortion. The ACOG 14 recommends continuing efforts to stop stigmatizing abortion and include it in medical training. They suggest that some measures are to include sexual education and therapeutic abortion in the curriculum of medical schools, as well as to improve exposure to residents for this procedure. Allen et al. 26 showed that the factor most strongly associated with whether the obstetrician-gynecologist provides abortion service was whether the provider was interested in training in it before starting residency. This is why it is vitally important to be able to expose medical students to these topics during their undergraduate studies. 26

Our study is the first to evaluate the perceptions of therapeutic abortion of a significant number of physicians from academic institutions in Peru. There are many barriers to training and access, and our study describes the most common and prevalent in Peru. For the development of our survey, we used tools previously used by other authors. In addition, we describe the different possible barrier areas such as leadership, resources, and support from other specialties, among others.

Our study also has limitations. The main limitation of our study is that the vast majority of the participants work in Lima, so it is possible that these results do not apply to different areas of Peru. Our study has a few limitations due to its design, such as the possibility of non-honest answers, different interpretations of the questions for each participant, and the possibility that some answers may be guided by the moral and/or religious position of the respondent regarding abortion treatment. Despite its limitations, the present study contributes significantly to knowledge about therapeutic abortion training in Latin America and plays a role in this important public health measure.

## Conclusion

Most doctors support therapeutic abortions and show interest in improving their skills; however, not all hospitals offer adequate training and education. During training, therapeutic abortion procedures are performed in a limited number. Also, lack of knowledge of the law and of institutional policies are common, making fear of ethical, legal, and religious repercussions the main barriers.
